# The impact of hospital-based and community based models of cerebral palsy rehabilitation: a quasi-experimental study

**DOI:** 10.1186/s12887-014-0301-8

**Published:** 2014-12-05

**Authors:** Jermaine M Dambi, Jennifer Jelsma

**Affiliations:** Research Fellow at University of Cape Town, Lecturer Department of Rehabilitation, College of Health Sciences, University of Zimbabwe, PO Box AV 178, Avondale, Harare, Zimbabwe; Division of Physiotherapy, Department of Health and Rehabilitation Sciences, Faculty of Health Sciences, University of Cape Town, Anzio Road, Observatory, Cape Town, South Africa

**Keywords:** Cerebral palsy, Community based rehabilitation, Institution based intervention, Rehabilitation, Zimbabwe

## Abstract

**Background:**

Cerebral palsy requires appropriate on-going rehabilitation intervention which should effectively meet the needs of both children and parents/care-givers. The provision of effective support is a challenge, particularly in resource constrained settings. A quasi-experimental pragmatic research design was used to compare the impact of two models of rehabilitation service delivery currently offered in Harare, Zimbabwe, an outreach-based programme and the other institution-based.

**Method:**

Questionnaires were distributed to 46 caregivers of children with cerebral palsy at baseline and after three months. Twenty children received rehabilitation services in a community setting and 26 received services as outpatients at a central hospital. The Gross Motor Function Measurement was used to assess functional change. The burden of care was measured using the Caregiver Strain Index, satisfaction with physiotherapy was assessed using the modified Medrisk satisfaction with physiotherapy services questionnaire and compliance was measured as the proportion met of the scheduled appointments.

**Results:**

Children receiving outreach-based treatment were significantly older than children in the institution-based group. Regression analysis revealed that, once age and level of severity were controlled for, children in the outreach-based treatment group improved their motor function 6% more than children receiving institution-based services.

There were no differences detected between the groups with regard to caregiver well-being and 51% of the caregivers reported signs consistent with clinical distress/depression. Most caregivers (83%) expressed that they were overwhelmed by the caregiving role and this increased with the chronicity of care. The financial burden of caregiver was predictive of caregiver strain.

Caregivers in the outreach-based group reported greater satisfaction with services and were more compliant (p < .001) as compared to recipients of institution-based services.

**Conclusion:**

Long term caregiving leads to strain in caregivers and there is a need to design interventions to alleviate the burden. The study was a pragmatic, quasi-experimental study thus causality cannot be inferred. However findings from this study suggest that the provision of care within a community setting as part of a well-structured outreach programme may be preferable method of service delivery within a resource-constrained context. It was associated with a greater improvement in functioning, greater satisfaction with services and better compliance.

## Background

Cerebral palsy (CP) is the most common paediatric neurological condition [1] and the principal cause of disability in children globally [2]. It is defined as “a group of disorders of the development of movement and posture, causing activity limitation, that are attributed to non-progressive disturbances that occurred in the developing fetal or infant brain. The motor disorders of cerebral palsy are often accompanied by disturbances of sensation, cognition, communication, perception, and/or behaviour, and/or by a seizure disorder” [3]. CP is a universal problem [2] with a global incidence of 2 to 3 cases per 1 000 births [4]. The exact prevalence in Zimbabwe is unknown: however, from extrapolated data, the incidence is similar and estimated at 1.55/1000 in rural areas and 3.3/1000 in urban areas [5].

Children with CP face multiple bio-psychosocial challenges [6,7]. This coupled to the fact that CP is a lifetime condition [2,3,8], may result in a considerable burden on caregivers of children with severe impairments, affecting their health and health related quality of life [9,10]. Rehabilitation treatment is an essential component [1,11] of the multi-disciplinary approach required to address the problems of children with CP and their families [8,12]. Researchers have not yet identified the most effective method of service delivery in terms of optimising the child’s potential and providing support to the caregiver, especially in low-income countries such as Zimbabwe. Issues such as accessibility and acceptability of services, compliance with training and efficacy of the intervention need to be considered when implementing any model of service delivery.

Different models of rehabilitation service delivery have been proposed in an attempt to provide affordable and appropriate support to people living with disabilities and these can be broadly classified as either institution–based (IB) or community based rehabilitation (CBR)/outreach-based (OR) approaches [13,14]. The roots of CBR can be traced back to the Declaration of Alma-Atta which led to the adoption of the global primary health care strategy by the World Health Organization (WHO). The aim of CBR was to provide primary health care and rehabilitation services to people with disabilities within their communities [15]. CBR has been in existence for more than 3 decades [15-18] yet little is known about its efficacy, effectiveness, relevance, appropriateness and sustainability as a service delivery model and public health strategy [19-21].

Zimbabwe utilizes a hybrid model of provision of rehabilitation services that is a blend of hospital-based and community-based approaches which are provided at district, provincial and central hospitals [22]. Unfortunately, a decade of socio-economic meltdown has resulted in deterioration of the health care delivery system [23]. At present, organization of rehabilitation services varies from institution to institution and is mainly governed by resource availability. Most institutions are now offering hospital-based services only. For instance, out of the six state central hospitals in Zimbabwe, only Harare Central Hospital (HCH) is at present running a consistent outreach program through its Children Rehabilitation Unit (CRU) [22]. The CRU is a specialized paediatric rehabilitation centre, which, for more than twenty years, has run a peri-urban, community-based outreach programme based on the WHO CBR model. Children and their care-givers (mostly mothers) gather in groups twice a month in community centres. Children receive some individual treatment from therapists and/or rehabilitation technicians (who have undergone two years of training). In addition, there are group activities and education sessions. In contrast, children in another high-density area of Harare, which is serviced by a different hospital, receive regular physiotherapy within an institutional out-patient setting. As the outreach programme relies on a certain amount of donor funding, it is somewhat more expensive to run [22].

There was a clear need to compare the two models of service delivery, not only to inform the on-going re-structuring of rehabilitation services in Zimbabwe [23], but to provide empirical evidence of the relative impact of CBR/outreach services as compared to institutionally-based rehabilitation [11].

The objectives of the study were therefore to compare the impact of the outreach (OR) and the institutionally based (IB) programs in terms of their impact on the children’s functioning, the strain on their caregivers, compliance with scheduled appointments and the overall satisfaction with the services received. It was anticipated that there would be little difference in the functional change between the two groups. The greater group interaction and support were expected to result in a greater decrease in the strain of the care-givers attending the outreach group. The satisfaction with services was expected to be greater in the outreach group as the service was brought to them and they did not need to travel far to get support for their children.

## Methods

A quasi-experimental design was used as it was a pragmatic trial and it was not possible to randomly assign children to one group or the other. The geographical location determines a child’s program allocation as the two areas are some distance apart, children and caregivers were thus obliged to attend one or other programme depending on their place of residence. A sample of convenience was drawn from the children treated under the OR program and IB CP clinics who attended the clinics during the first four weeks of the study.

The children had to have received a diagnosis of CP according to their patient notes. They were to be between 0.5 to 12 years of age as the Gross Motor Function Measure (GMFM) has good content and face validity for children in the age range 0.5 to 13 years [24] and the discharge age for the CRU Outreach program is 12 years. No participant was recruited if they were scheduled for surgery or if they had any other significant medical and nutritional problems or other clinical factors that might have biased the rehabilitation program [25,26]. Children who had co-morbid neurological conditions e.g. Spinal Bifida or who were receiving other forms of therapeutic interventions such as private physiotherapy were similarly excluded.

The burden of care as measured by the Caregiver Strain Index (CSI) [27] was one of the major variables under scrutiny. Assuming mean CSI scores of 7 and 9 (SD = 2) for both groups [28] at the conclusion of the study period, the expected minimal number of cases per group was 16 (alpha = .05, power = 95%). Oversampling was done to counteract effects of attrition due to e.g. sickness and non-compliance.

### Instrumentation

The Gross Motor Function Measure −66 item version (GMFM-66) is a condition specific and widely used, standardized and validated ordinal scale which measures changes in motor function in children with CP [24,29,30]. Functional prognosis is dependent on level of severity and this we measured using the Gross Motor Function Classification System (GMFCS) which is a valid and reliable tool [24,31]. This classifies severity on a 5-level ordinal scale, with children in level one being least affected and level five being more severely affected and functionally dependent [31]. The Caregiver Strain Index [32] and Medrisk Instrument for Measuring Patient Satisfaction with Physical Therapy Care (MRPS), [33] have been reported to be valid and reliable tools in measuring the burden of care and satisfaction with services respectively [27,33]. The tools were translated into the native language, Shona, using a backward-forward approach. The tools were then validated on a group of caregivers, n = 20 of children with CP receiving outpatient services at CRU who were not part of the main study. The caregivers completed the questionnaires and were requested to give comments on the appropriateness, validity and clarity of the tools. After feedback, the tools were re-administered after a week in order to assess the internal consistency (Cronbach’s alpha = .78), validity and reliability (r = .82) of the tools, all of which were found to be acceptable.

### Intervention

The children and parents (mostly mothers) in the OR arm gathered in groups twice a month in community centres. In the IB arm, the frequency of appointments was variable and was dependent on the discretion of the treating therapists. In both arms, children received some individual face to face treatment from therapists. In addition, the OR arm received group activities, where caregivers were requested to demonstrate home exercise programs to other caregivers as well as sharing the challenges and achievements of caregiving. Additionally, the OR arm received educational sessions on the aetiology, management of CP and ways of coping with the associated burden of care. They were provided with light refreshment after therapy sessions and were given the option to participate in caregiver support group activities such as joint income generation projects. The OR programme receives donor funding and employs more rehabilitation professionals which improves the therapist/child ratio. In addition, allowances paid to professionals for every outreach outing makes it more expensive to run [22].

### Procedure

A pilot study was done to determine the intra-rater reliability of the GMFCS and GMFM-66 scoring as well as refining data collection procedures (see above). Caregivers were then recruited by the research team over four consecutive weeks. Caregivers were approached as they were awaiting services or after their children were treated. Once informed consent had been obtained, CSI questionnaires were distributed to caregivers which were self-administered. The principal researcher then documented the motor function scores of children with CP using the GMFM-66 and the GMFCS. It would have been difficult to transport participants to a neutral venue so all assessments were done at usual treatment settings and the usual treatment days to avoid inconveniencing the caregivers. Consequently blinding to group membership was not possible. The compliance with scheduled appointments was captured throughout the study. Twelve weeks later, the same procedure was followed in scoring the CSI, GMFM-66 scores and additionally the modified MRPS questionnaire was applied. Both groups were provided with snacks and drinks after data collection procedures.

### Ethical considerations

Ethical approval was granted by the University of Cape Town (ref 109/2012) and the Medical Research Council of Zimbabwe (MRCZ/B/333). Consent was sought from caregivers, rehabilitation professionals and verbal assent was requested from children who could communicate (n = 5). Fifteen caregivers refused consent. Caregivers were assigned identity numbers to preserve confidentiality and only the principal researcher had access to the collected raw data which was kept in a safe locker. Both groups of participants were treated equally to achieve social justice.

### Data analysis

Statistical analysis was performed using STATISTICA version 10. We used an alpha level of 0.05 for all statistical tests. Analysis was per protocol. As most of the data were non-parametric, the Mann–Whitney U, chi-squared and Fishers’ Exact tests were used to compare results between the two groups in terms of the difference in demographics, MRPS and CSI. The scores on the GMFM-66 were transformed into interval data using the Gross Motor Ability Estimator (GMAE-2) Scoring Software for the GMFM [34] which is a software package for scoring the Gross Motor Function Measure (GMFM) based on item response theory. A one way ANOVA was used to compute differences in GMFM-66 and CSI scores at different times.

As age was significantly different between the two groups and there were more severely affected children in the community based treatment group, regression analysis was done to establish which factors predicted the amount of change in the GMFM Score. Dummy variables were created for the categorical variable of the group and the ordinal variable of GMFCS was dichotomised into levels mild/moderate (I, II and III) and severe (IV and V). Residual analysis was performed and children who had residual scores of more than 2.5 SD from the mean were excluded.

## Results

### Demographic and medical characteristics

**Figure 1:**
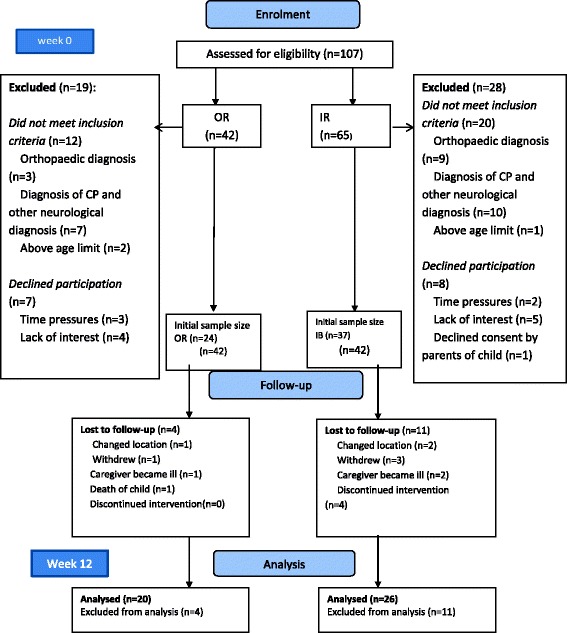
**Flow chart of the study.** 107 potential participants were approached, of which 28 did not meet the inclusion criterion. Of these, 15 were lost to follow up given a final sample size of 46 for data analysis.

A total of 107 potential participants were approached for recruitment into the study, of these, 42 were from OR and 65 from IB. As can be seen in Figure 1, 32 did not meet the inclusion criteria or declined participation (15). A further four in the OR group and 11 in the IB were lost to follow-up for different reasons. Ultimately 20 in the OR and 26 children in the IB groups completed the study. Demographic information on the 46 dyads of caregiver and child with CP who participated are presented in Table 1. Children receiving IB treatment were significantly younger than those in the OR group, (12 as opposed to 44 months). However, the two groups were comparable in terms of the socio-demographics of both children and caregivers at baseline. In the IB group 38% of the children were in the most severe levels of the GMFCS, compared to 50% in the OR group, however the proportions in each level were not significantly associated with group.

**Table 1:** **Study population demographic characteristics, N = 46**

		**OR (n = 20)**	**IB (n = 26)**	**Total**	**Statistic**	**p-value**
		**n**	**n**	**n (%)**		
Gender	Males	11	14	25 (54)	*χ* ^2^ = 0.049	0.825
	Females	9	12	21 (46)	df = 1*	
Mean age of children in months (SD)		44 (49)	12 (7)	26 (36)	U = 170.0, Z = −1.928	0.047
GMFCS Level	I	5	8	13(28)	U = 0.790	0.448
	II	3	4	7 (15)	Z = 0.429	
	III	2	4	6 (13)		
	IV	2	2	4 (9)		
	V	8	8	16 (35)		
CP type	Spastic	16	21	37 (80)	*χ* ^2^ = 0.0	0.948
					df = 3	
	Athetoid/dyskinetic	3	2	5 (11)		
	Ataxic	1	1	2 (4)		
	Mixed	0	2	2 (4)		
Mean caregiver age (SD) in years		33 (12)	28 (5)	30.4 (9.2)	U = 192.00, Z = 1.496	0.134
Relationship to child	Mother	16	22	38 (83)	*χ* ^2^ = .710	0.701
					df = 2	
	Grandmother	2	3	5 (11)		
	Sibling	2	1	3 (7)		
Caregiver	Primary	2	2	4(9)	*χ* ^2^ = 4.268	0.371
					df = 3	
Educational level	Secondary	13	17	30 (65)		
	Tertiary	4	5	9 (20)		
	None	1	2	3 (7)		
Caregiver	Unemployed	12	16	28 (61)	*χ* ^2^ = 0.802	0.67
					df = 2	
Employment status	Informally employed	7	7	14 (30)		
	Formally employed	1	3	4 (9)		

*- With Yates correction of continuity.

### Treatment sessions and compliance

The therapist hour’s ratio was calculated by dividing the product of number of therapists and total number hours of therapy provided by total number of children treated over the study period. As can be seen in Table 2, they were no statistically significant differences in terms of the organization of treatment sessions, *χ*^2^ = 0.711, df = 1, p = 0.399 and children in the OR group received a significantly higher amount of therapy time, t(43) = 3.19, p = 0.003.

**Table 2:** **Treatment sessions details for the study duration**

		**OR**	**IB**	**Statistic**	**p-value**
Type of treatment rendered	Individual therapy sessions	3	3	*χ* ^2^ = 0.711	0.399
				df = 1*	
	Health promotional talks	6	3		
Therapist hours ratio	Mean (SD)	0.29 (.07)	0.21 (.10)	t(43) = 3.19	0.003
	Median	0.30	0.37		
	Range	0.20-0.40	0.27-0.54		

*- With Yates correction of continuity.

Caregivers in the OR group were expected to attend every two weeks and in the IB group, caregivers were given a variable number of appointments; this is illustrated in Table 3.

**Table 3:** **Frequency of appointments for the study duration, N = 46**

**Variable**	**Attribute**	**OR**	**IB**
Met appointments	Mean (SD)	5.6 (0.7)	3.8 (2.6)
	Median	6	3
	Range	4-6	1-9
Scheduled appointments	Mean (SD)	6 (0)	5.1 (3.1)
	Median	6	5
	Range	-	1-10

The percentage compliance was calculated by dividing the number of attendances by the maximum number of attendances possible. The mean percentage compliance was significantly greater in the OR group: 93.3% (median = 100, range: 67–100) for the OR group and 72.8% (median = 72.5, range: 33–100) for the IB group, (Z = −3.56, p < .001).

### Impact on function

The GMFM-66 scores over time (Table 4) were compared and whereas there were no between group differences detected, the improvement over time for both groups combined was significant.

**Table 4:** **Change in GMFM 66 scores over three months, n = 46**

	**IB**	**0R**	**Total**	**Statistic**	**p-value**	**Confidence-95%**	**Confidence + 95%**
Baseline, Mean (SD)	42.9 (8.1)	39.7 (14.4)	41.5 (11.2)	t = 2.04	0.047	−5.20	−0.04
Three months, Mean (SD)	43.5 (9.0)	44.9 (19.8)	44.1 (14.5)	df = 45			

The regression model (Table 5) with the change in GMFM-66 scores as dependent variable accounted for about a quarter of the variance (adjusted R^2^ = .27) after residual analysis resulted in the scores of two children being removed. The results indicate that, once age and category were controlled for, children in the OR group improved 2.49 points more on the GMFM-66 than children receiving IB services. This equates to approximately a 6% difference in improvement (2.49/41.5 at baseline). Children who were less severely disabled showed 1.96 points more improvement and for each month of age, older children showed .02 less improvement

**Table 5:** **Predictors of the change in GMFM-66 scores over three months, n = 46**

	**Amount of change - b**	**Standard error of b**	**t(41)**	**p-value**
Intercept	−0.39	0.61	−0.6	0.526
OR group	2.49	0.75	3.3	0.002
Minimal severity	1.96	0.67	2.9	0.005
Age (months)	−0.02	0.01	−2.3	0.029

### Impact on caregivers

The majority of both groups reported an impact on inconvenience, physical strain, confining, family adjustments; personal plans and work adjustments (Table 6). The greatest number reported problems with financial strain and feeling overwhelmed.

**Table 6:** **Responses to the caregiver strain index (n = 46)**

**Number reporting problems**	**Baseline**			**At three months**		
	**OR**	**IB**	**Total**	**OR**	**IB**	**Total**
	**n**	**n**	**n (%)**	**n**	**n**	**n (%)**
Sleep	3	9	12 (26)	2	9	11(24)
Inconvenient	8	16	24 (52)	10	15	25 (54)
Physical strain	10	19	29 (63)	10	18	28 (61)
Confining	12	13	25 (54)	9	13	22 (48)
Family adjustments	10	16	26 (57)	7	14	21 (46)
Personal plans	11	19	30 (65)	11	21	32 (70)
Emotional adjustments	7	16	23 (50)	7	16	23 (50)
Upsetting behaviour	8	7	15 (33)	6	5	11 (24)
Has changed	6	6	12 (26)	7	5	12 (26)
Work adjustments	11	15	26 (57)	11	13	24 (52)
Financial strain	14	15	29 (63)	17	17	34 (74)
Overwhelmed	14	22	36 (78)	16	24	40 (87)

Further, the caregivers experienced a high burden of care (Table 7) and 50% (n = 23) of the caregivers had scores greater than or equal to seven which is the cut-off point for clinical distress/depression [32].

**Table 7:** **CSI scores comparison at baseline and at three months, (n = 46)**

	**At baseline**					**At three months**				
	**OR**	**IB**	**Total**	**Statistic**	**p-value**	**OR**	**IB**	**Total**	**Statistic**	**p-value**
Median	5.5	7	6.5	U = 204	0.219	6	7	7.0	U = 220	0.381
				Z = 1.230					Z = 0.87	
	n	n	n (%)			n	n	n (%)		
Normal (0–6)	12	11	23(50)	*χ* ^2^ = .796	0.372	11	11	22 (48)	*χ* ^2^ = .31	0.578
Clinical distress (7–12)	8	15	23(50)			9	15	24 (52)		

The sign test indicated that there were no changes in CSI score over the course of the study (p = 1.0). There were also no differences in the median scores between the two groups or in the proportion reporting clinical distress (score greater than seven) either at baseline (p = .385) or three months (p = .221).

### Satisfaction with services

As shown in Table 8, caregivers in the OR group reported greater satisfaction with services and statistically significant differences were found in all domains apart from the registration process, comfort of the waiting area and being treated with respect.

**Table 8:** **Responses to the satisfaction with services (Medrisk) questionnaire (n = 46)**

**Satisfaction domain**	**Group**	**Strongly disagree n**	**Disagree**	**Neutral**	**Agree**	**Strongly agree**	**Z adj**	**p value**
			**n**	**n**	**n**	**n**		
Registration process	OR	0	0	1	7	12	1.4	0.221
	IB	0	0	2	14	10		
Comfort of waiting	OR	1	0	5	8	6	1.1	0.317
Area	IB	0	1	10	11	4		
Time therapist spends	OR	1	1	2	4	12	3.1	0.002
with child	IB	0	4	10	10	2		
Amount of explanations	OR	0	0	1	4	15	2.9	0.006
given by therapist	IB	0	5	4	8	9		
Being treated with	OR	0	1	1	5	13	1.1	0.327
respect	IB	0	1	2	11	12		
Having concerns	OR	0	0	0	4	16	3.6	0.001
listened to	IB	0	1	10	7	8		
Having all questions	OR	0	0	2	3	15	2.9	0.006
answered	IB	0	2	7	9	8		
Being given future	OR	0	0	0	5	15	3.8	.001
advice	IB	1	5	5	9	6		
Receiving instructions on home exercise	OR	0	1	2	2	15	2.3	0.035
program	IB	2	3	4	6	11		
Overall satisfaction	OR	0	1	1	5	13	3	0.004
	IB	4	3	6	6	7		
If they will return for	OR	0	0	2	4	14	2.3	0.037
future services	IB	0	1	8	7	10		

## Discussion and conclusions

The objectives of the study were to compare the impact of the outreach (OR) and the institutionally based (IB) programs in terms of their impact on the children’s functioning, the strain on their caregivers, compliance with scheduled appointments and the overall satisfaction with the services received. It should be noted that the “entire package”, which encompassed the location (community or institutional based), the increased training and experience of the OR personnel, the structure of the therapy sessions and the provision of refreshments during the OR sessions were compared. It was not possible, using this research design, to isolate which components of the programmes resulted in the differences seen. The results of the study indicate that in several respects the OR programme was superior to the IB programme. The sample appeared to be representative of children with CP in that the majority had spastic type CP (80%) which is the most common variant of CP as reportedly accounts for 80–83% of cases [11,35-37]. The spread across the different GMFCS levels was similar to a large scale study in Canada, which reported 42% of the children were severely affected (Levels IV and V) compared to 44% in this study [31]. The predominance of males in the sample has also been reported in other samples of children with CP [6,38,39]. It would therefor appear that the children in this study were representative of most samples of children with CP.

A problem with quasi-experimental studies is that there may be confounding variables that may bias the results of the study. In this case, there were no differences found between the participants in the OR group and the IB group in terms of demography or nature of their impairment. The differences that were noted, that the OR children were older and more were severely affected (although not statistically so), would have introduced bias into a randomised trial. However, in this pragmatic trial it was an indication of the strength of the OR intervention in that older, more severely affected children were still being brought in for treatment. It has been reported that older and more severely affected children might respond less to interventions, [12]. It was thus necessary to control for these factors by doing regression analysis. Children in the OR group showed greater improvement and several factors can account for this difference. Firstly, some of the rehabilitation workers in the OR group are based in a specialist unit and have developed skills in child treatment whereas the IB rehabilitation professionals are responsible for treatment across a wider spectrum of conditions and ages. Secondly, the lower child to therapist ratio in the OR group ensures ample time for treatment and demonstration of techniques to caregivers. Thirdly, continuity of care in the OR group,and the inherent good therapist-child relationship may have led to increase in-treatment adherence and this may have enhanced treatment efficacy [40].

The situation in Zimbabwe is typical of a resource constrained country in that children with severe CP are not necessarily catered for within institutions or special schools. The response to therapy might be different in children who have had on-going intensive rehabilitation within specialised centres. In addition, parents who have had access to sophisticated services may not demonstrate the same degree of satisfaction with the type of service provided. The results of the study may therefore only be of relevance to low and middle income countries.

As the children in the community based group were older and higher proportions were in GMFCS Levels IV and V, their caregivers might be expected to report greater strain. This was not the case, which might indicate that the outreach based intervention mitigated the impact of severity and chronicity of care to a certain extent. This hypothesis however, needs to be empirically tested. Alternatively, it may be that parents of younger children are in earlier phases of ‘grief’ in response to having a child with a disability which may dissipate over time to some extent [41]. It is clear that the care-givers are in need of additional support, particularly financial and emotional as there are no disability grants in Zimbabwe.

Caregivers in the OR group seemed to be more satisfied with services and were more compliant as compared to recipients of IB services. It is essential to evaluate patient satisfaction with services delivery as satisfaction is related to treatment compliance and outcomes [33,42]. Services in the OR group were provided every fortnight and this could have enhanced satisfaction and compliance with services. Furthermore, consistent booking schedules have been demonstrated to affect the levels of compliance and satisfaction with services [43-45]. Additionally, provision of services within the recipients’ communities, a more natural environment, negates the need for transportation costs and adapted transportation (which may not be available in low resourced settings). It also results in an increased amount of social support. All of which have been identified cited as enhancers to satisfaction and compliance [46-50].

Lack of knowledge of the child’s impairment can lead to non-compliance [51]. As caregivers in the OR group would have attended CP workshops prior to joining the outreach group, it was expected that they would have been more knowledgeable about CP. This might have enhanced compliance and satisfaction with the explanations given by therapists on CP and its treatment. This hypothesis was not tested however and a weakness of the study was that the amount of information that the caregivers had regarding CP was not compared between the groups.

The IB group had a higher patient to therapist ratio; this inherently leads to time pressure during treatment sessions. Time pressure may lead to decreased in-treatment adherence, less satisfaction with explanations and therapy given. This may have accounted for the lower compliance and satisfaction in the IB group. The nature of the patient/practitioner relationship also affects the extent of compliance and satisfaction [51]. Further, the absence of continuity of care in the IB group, might also have accounted for lower rate of compliance and satisfaction.

Research on the effect of treatment frequency has yielded inconclusive evidence [12,40,43]. However, a study by Christiansen & Lange, [43] suggests that intermittent frequency is equally efficacious when compared to continuous dosage. Therefore, evidence from our findings suggests that a two week gap may be tolerable for caregivers and may result in equal gains in functional outcome.

Results from this study need to be interpreted with caution as children and caregivers had had interventions for varying lengths of time and changes in the outcome measures might have taken place prior to the study. Secondly, methodological weakness of the study design and the lack of randomisation and blinding of the assessor may limit the generalizability of our findings. Thirdly, there was a difference in expertise and clinical experience for therapists in the comparison groups with those in the OR arm more experienced and this could have introduced bias.

In conclusion, long term caregiving leads to strain in caregivers and there is a need to design individualized interventions to alleviate the burden on caregivers as it may ultimately affect the child’s functional prognosis and health outcomes. The study was a pragmatic, quasi-experimental study, which by its nature cannot lead to causal inference. However findings from this study suggest that the provision of care within a community setting as part of a well-structured OR programme may be preferable. It was associated with a greater improvement in functioning, greater satisfaction with services and better compliance. In addition, care-givers continued to bring in older children for therapy, which was encouraging. It is therefore suggested that may be the preferred method of service delivery. Further research is needed however, to cost the methods of service delivery in order to determine the cost of transferring the management of children with CP from institutions to the community.

## Abbreviations

CBR: Community based rehabilitation
CP: Cerebral palsy
CRU: Children rehabilitation unit
CSI: Caregiver strain index
GMFCS: Gross motor function classification system
GMFM: Gross motor function measurement
GMFM-66: Gross motor function measure −66
HCH: Harare central hospital
HRQoL: Health- related quality of life
IB: Institution–based
MOHCWZ: Ministry of health and child welfare Zimbabwe
MRPS: Medrisk instrument for measuring patient satisfaction with physical therapy care
OR: Outreach-based
WHO: World Health Organization
